# The Effect of Gypsum on the Self-Hardening of Sapropel in Thermal Insulating Wood Chips Composite

**DOI:** 10.3390/ma18102217

**Published:** 2025-05-11

**Authors:** Jurga Šeputytė-Jucikė, Sigitas Vėjelis, Saulius Vaitkus, Agnė Kairytė, Arūnas Kremensas, Giedrius Balčiūnas

**Affiliations:** Building Materials Institute, Faculty of Civil Engineering, Vilnius Gediminas Technical University, Linkmenų str. 28, LT-08217 Vilnius, Lithuania; jurga.seputyte-jucike@vilniustech.lt (J.Š.-J.); saulius.vaitkus@vilniustech.lt (S.V.); agne.kairyte@vilniustech.lt (A.K.); arunas.kremensas@vilniustech.lt (A.K.); giedrius.balciunas@vilniustech.lt (G.B.)

**Keywords:** sapropel, gypsum, hydrated lime, wood chips, mechanical performance, thermal conductivity, structure analysis

## Abstract

An environmentally friendly and rational way of using wood waste is by introducing it into composite compositions. This paper investigates the use of wood chips from 10 to 60% for creating a thermal insulation composite. Prepared wood chips of various fractions were mixed with the sapropel/gypsum mixture. The composite with wood chips and a mixed sapropel/gypsum binder was hardened without thermal curing. Gypsum was added to absorb water from the sapropel and to give the composite its initial strength. Hydrated lime was used to improve the compressive stress of the binding material. The composite density varied from 400 to 1050 kg/m^3^, thermal conductivity varied from 0.0912 to 0.193 W/(m·K), and compressive stress varied from 0.2 to 7.9 MPa. The density of the composite and the studied properties depended on three factors: the ratio of sapropel to gypsum, the ratio of wood chips to binder, and the level of compaction. The content of sapropel/gypsum varied from 10 to 90%, the ratio of wood chips to binder varied from 0.5 to 1.5, and the compaction level varied from 16 to 40% according to the initial height of the mould. The main characteristics of the prepared composites with different sapropel/gypsum and wood chip ratios were determined. The density, compressive stress, and thermal conductivity results were statistically analysed.

## 1. Introduction

Over the past two decades, ecological composites have received particular attention in various industries [1,2,3]. The use of ecological materials that are recyclable, biodegradable, and renewable is part of the effort to reduce the environmental impact of petroleum-based materials. Entirely biodegradable green composites are biocomposites produced by combining biofibres and resins from raw materials from renewable agricultural and forestry industries [4]. These composites can be recycled after their useful life without causing damage to the environment [5].

Agriculture and forestry provide large amounts and a wide variety of raw materials that can be used as fillers in lightweight composites. Straw from various crops or their processing waste is derived from agriculture [6,7,8,9]. Stems from non-cultivated plants can also be used [10,11,12]. Forestry produces large amounts of wood waste generated during the processing of raw wood material [13,14,15,16]. In addition, wood waste can also be obtained from the processing of multipurpose wood products already used at the end of their life cycle [17,18]. The scope of application of the aforementioned organic fillers is extensive—they are intended for various construction composites, particle boards, mats, bricks, and concrete mixtures.

Lignin, protein, and starch are often identified as the main types of natural biomass binders [19,20,21,22]. Their uses include not only various building composites and boards but also other areas, fuel briquettes, a binder in printed wiring boards, abrasive tools, epoxy asphalts, epoxy wood composites, 3D printing, adhesive hydrogels, soil suppressants, lignocellulosic paper, and coatings. Although several binders have been developed from renewable resources, not all are widely used for various reasons. These reasons are usually insufficient performance or high price. Furthermore, most binders are cured at high temperatures [23,24,25].

Sapropel has been studied as a binding material in several scientific works [26,27,28,29,30]. Depending on the formation method, the sapropel can be organic or mineral. Only organic sapropel is used as a binder because it has binding properties. The typical chemical composition of an organic sapropel is as follows: C (54.0%), N (1.1%), O (37.2%), Si (4.1%), S (0.3%), and other elements (3.3%) [31]. Obuka [32] states that sapropel is beneficial because it consumes little energy and emits little CO_2_, making it suitable for use as an eco-friendly building material. Sapropel takes a long time to harden naturally, so it needs to be thermally treated, or another material needs to be applied that accelerates hardening.

In recent years, gypsum has been increasingly used for its reasonable thermal, strength, and acoustic properties; environmental friendliness; and fast hardening [33]. The main disadvantages of gypsum are its high density, brittleness [34,35], and low resistance to water effects [36]. Using gypsum binding material with fillers and appropriate additives more or less eliminates these disadvantages [37,38]. In addition, gypsum has an important characteristic—good fire resistance [39].

Authors [33,40] note that interest in lightweight composites from natural fibres and gypsum binder is constantly growing.

Morales-Conde et al. [41] examined 50 compositions of composites made with gypsum binder, wood chips, and sawdust. The authors found that increasing the amount of wood waste significantly decreases the density and thermal conductivity of the composite but decreases the strength characteristics. When 40% of the wood chips and sawdust were used, the bending strength decreased by 61 and 65%, and the compressive strength decreased by 71 and 78%, respectively. The researchers managed to improve the strength properties using glass fibre. In their study, the authors selected the water/gypsum ratio based on the amount of wood waste used—a ratio of 0.55 was selected using 2.5, 5, and 10% of wood waste. This ratio was 0.80 when 20% waste was used and 1.25 when 40% waste was used.

Several scientific studies show that reducing the amount of gypsum in the composite decreases its strength indicators [37,42,43]. The decrease in strength properties is related to the number of fillers and their origin. Pedreno-Rojas et al. [43] found that with organic fillers, compressive strength decreases by almost 3.2 times when the number of organic fillers is increased from 5 to 40% and by almost 1.2 times when using polymeric fillers.

In another study [44], Shiroma et al. developed a gypsum/wood plaster. The researchers used a water/gypsum ratio of 0.65 for this plaster, and the amount of wood particles per mass was 5, 10, and 15%. Additionally, the researchers treated the wood particles with room-temperature water and 80 °C water to reduce the amount of solutes that interfere with the crystallisation of the gypsum plaster. Guedri et al. [33] treated natural fibres with Na(OH) for a composite with gypsum plaster and found that the fibres’ surface became rough, and the strength indicators of the composite were improved. Wu et al. [45] treated wheat straw with sodium hydroxide (NaOH) solution and silane coupling agent (KH-602) before manufacturing the composites. The internal bond strength of such a composite was 173.9% higher than that of conventional gypsum board because the chemically treated wheat straw formed better contact areas.

In another study [42], Mutuk et al. investigated the influence of natural fibres, hemp, and banana on composites’ mechanical, thermal, and structural characteristics with gypsum binder. Scientists have found that even 1% of natural fibres significantly influence the composites’ properties. When 1% of hemp and banana fibres are used, compressive strength decreases from 10.9 to 6.83 MPa, but the thermal conductivity also decreases significantly, from 0.287 to 0.176 W/(m·K). The authors found that fine and large pores are formed in the composite because of the fibres, leading to changes in their properties. Vavrinova et al. [39] used different grades of gypsum and chopped wheat straw. Scientists indicate that after using 5% of straw, the compressive strength decreased by as much as 80.27%, and the thermal conductivity decreased very little—to 0.332 W/(m·K).

The literature analysis shows that gypsum/sapropel binder has not been used to develop building materials. Therefore, this work focuses on developing and investigating this new binder by evaluating the strength and thermal properties of the composite. Using gypsum and sapropel binder and determining their appropriate ratio, we assume that the resulting binder will not require thermal treatment and will harden naturally. We predict that the hardening time will be shorter than that of other classical binders and will be suitable for natural production conditions.

This work aims to use the highest possible amount of wood chips and sapropel to achieve the lowest possible thermal conductivity without significantly degrading the strength characteristics of the composite. It also attempts to combine two binding materials, gypsum and sapropel. Gypsum was added to absorb water from the sapropel and to give the composite its initial strength. The gypsum/sapropel binder was selected in different ratios, and ready mixtures were compacted or compressed to the intended limit. Hydrated lime was used to improve the properties of the binding material.

## 2. Materials and Methods

### 2.1. Materials Used in Experiments

Sapropel was taken by the company JSC EcoLotus (Alytus, Lithuania) from the bottom layer of a lake. The sapropel was excavated mechanically with a dredger. The main specifications of the dredger are a general pump capacity of up to 100 m^3^/h, an operating depth of up to 35 m, and a horizontal transportation distance of up to 1000 m [46]. A total of 100 L of sapropel was used for the experiments. The characteristics of the sapropel used in the study are presented in Table 1.

For the tests, building gypsum “Baugips” (SIA Knauf, Saurieši, Latvia) was used (Table 2). The water content of the sapropel was determined according to EN 12570 [47]. EN 13820 [48] was applied to determine the organic matter content. The chemical composition of the sapropel used was as follows: C (47.2%), N (3.8%), O (36.1%), Si (7.43%), S (1.34%), Ca (1.59%), and other elements (2.54%).

Hydrated lime (calcium lime (CL)) was used with more than 90% CaO + MgO (CL 90 S according to EN 459-1 [49]); available or active lime content, in wt.%, was ≥80; MgO content, in wt.%, was ≤5; and compressive strength when mixed with sand varied in the range of 2.4–17.2 MPa. The amount added ranged from 5 to 20% of the amount of gypsum.

Pine wood chips with a size of 0–20 mm (Table 3) and 87 kg/m^3^ density, purchased from AB Lytagra, Vilnius, Lithuania, were used as the composite filler. The wood chips were chemically treated with a 10% sodium carbonate solution to improve adhesion to the binder. The characteristics of the sodium carbonate were a mass fraction of sodium carbonate of 99.8%, and a bulk density of 0.59 g/cm^3^.

The obtained solution was poured into a metal pot with wood waste, covered with water, and boiled for an hour. Then, it was set to cool for 23 h. The solution then passed through a metal sieve, and the pot was filled with water again. After repeating the procedure six times, the wood chips were left to drain on a metal sieve for 24 h and then dried to a constant mass.

### 2.2. Specimen Preparation

The samples were prepared at a temperature of 23 ± 5 °C and a relative humidity of 50 ± 5%. The compositions of the mixtures are presented in Table 4, Table 5 and Table 6.

The mixtures were mixed using a laboratory mobile vertical mixer Apex ST2 (Wagner S.p.a, Valmadrera, Italy) in a round 50 l plastic container. The mixer speed was 280 rpm for the whole mixing period. The total duration of mixing was 30 s. After mixing, the entire mixture was added and compacted in the mould in 40 s. Some of the specimens were compacted manually, while other parts of the specimens were compressed under a predetermined load. The compression level for different amounts of wood chips was determined experimentally. Table 6 presents the compositions of specimens with different ratios of gypsum to sapropel for determining the compressive strength of the hardened binder. Table 5 presents the compositions of specimens with different ratios of wood chips to binder for determining the compressive strength of the composite.

Table 6 presents the compositions of samples with different wood chips to binder ratios for thermal conductivity tests and evaluates the influence of sapropel on determining the composite’s thermal conductivity.

### 2.3. Test Methods

Before testing, all specimens were dried at 105 °C temperature in an oven to a constant weight.

The density tests were conducted according to EN ISO 29470 [50]. The specimens’ dimensions were 50 × 50 × 50 mm^3^.

Compression tests were performed according to the requirements of the EN ISO 29469 standard [51]. The requirements of this standard apply to thermal insulation materials and are characterised by exceptional indicators: the compressive strength is evaluated after reaching the maximum load or a specific load at 10% deformation. This work determines the compressive stress at 10% strain for the composites because the specimens usually do not undergo a sudden visible disintegration. However, the compaction of the specimen occurs. Specimens with dimensions of 50 × 50 × 50 mm^3^ were prepared for compression tests. Four specimens of each composition were prepared. Hounsfield Qmat–10 (England) universal testing machine was used for the tests. During the tests, the loading speed was constant at 0.1 d mm/min, where d is the thickness of the specimen.

The thermal conductivity was measured based on the EN 12664 standard [52]. The FOX 304 heat flow meter (TA Instruments, New Castle, DE, USA) with active edge protection and software was used for the tests. Thermal conductivity measurements were performed in an average temperature environment of 10 °C. Three specimens with dimensions of 300 × 300 × 50 mm^3^ were prepared to measure each composition. Before testing, all specimens were dried to constant weight.

The structure of the composites was analysed with a scanning electron microscope (SEM) (Carl Zeiss SMT GmbH, Oberkochen, Germany). The SEM instrument’s magnification ranged from 1 to 1,000,000 times, and the magnification range used during the experiment was from 100 to 10,000 times. Before structural analysis, all samples were prepared sputter-coated with a thin carbon layer.

## 3. Results and Discussion

### 3.1. Analysis of the Compressive Stress of Binding Material

**Figure 1:**
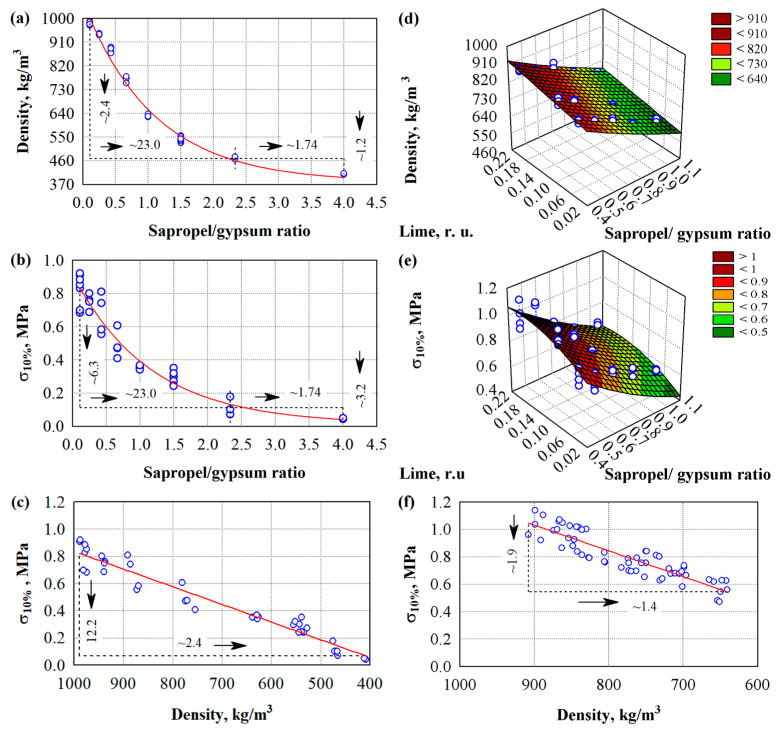
Influence of the composition of the binding material on the density and compressive stress of hardened specimens: (**a**–**c**) results of hardened binding material with different sapropel/gypsum ratios (compacted manually); (**d**–**f**) results of hardened binding material with different sapropel/gypsum/lime ratios (compacted manually); ○—experimental data; ─── average line.

In the initial stage, specimens with different ratios of sapropel and gypsum were prepared. The influence of the sapropel/gypsum ratio on density and compressive stress was evaluated from the prepared specimens. The graphical expression of the obtained results is presented in Figure 1a–c. Mathematical–statistical analysis was applied to evaluate the experimental results: regression and variance analysis [53]. In Figure 1a, it can be seen that the density of the hardened binding material decreases significantly with an increase in the sapropel/gypsum ratio.

The minimum density of the hardened binder is around 400 kg/m^3^, and the maximum is around 1000 kg/m^3^. When the amount of sapropel in the binder is increased and the density of the binder is decreased, the compressive stress ($\sigma_{10\%})$ also decreases significantly (Figure 1b,c). The most significant decrease in material density and compressive stress is observed when the sapropel/gypsum ratio increases from ~0.1 to 2.3. Furthermore, it was determined that when more than 50% of the sapropel was used, the final setting time of the binding material was extended from several hours to several days, so in further studies, more than 50% of the sapropel was not used. To improve the properties of the binding material, we used hydrated lime (Figure 1d,e). For the formation of the composite, according to the results of the strength characteristics, the ratio of sapropel/gypsum binder was selected as 0.43 (Figure 1).

After performing the mathematical–statistical analysis of the experimental results, regression dependencies were obtained, which are presented in Table 7. It should be emphasised that in Table 8 and Equations (2)–(6), the coefficients of determination of the regression equations range from 0.794 to 0.989. The resulting regression equations can be reliably applied to predict the properties obtained from known values.

Statistical data analysis showed that at the same material density of ~650 kg/m^3^ (Figure 1a,f), the difference in compressive stress between sapropel/gypsum and sapropel/gypsum/lime reaches ~46.7%, and for a density of ~900 kg/m^3^ (Figure 1a,f), the difference in compressive stress between sapropel/gypsum and sapropel/gypsum/lime reaches ~50.4%. It can be assumed that adding lime to the sapropel/gypsum mixture significantly increases compressive stress. We determined that its rational amount is 10% of the amount of gypsum. A higher amount of lime has no or negligible effect on stress. The use of lime in various mixtures is often described. Elert et al. [54] conducted a detailed analysis of the effect of lime on gypsum mortars.

They concluded that even small lime additions reduced strength but significantly improved weathering resistance. The lime content used in the work ranged from 5 to 95%. The authors found that gypsum/lime mortar had a 42–57% porosity, but no clear correlation with the lime content could be established. Naciri et al. [55] found that increasing the gypsum content in gypsum/lime/brick powder mortar improves strength indicators. Scientists have found that the strength indicators improve due to the lime and brick powder reaction. In our case, the increase in strength properties may have been due to the interaction of sapropel and lime particles. Sapropel contains humic and fulvic acids, which are complex organic polymers with acidic functional groups (e.g., carboxyl and phenolic hydroxyls). Hydrated lime, being a strong base, can react with these acidic groups through neutralisation reactions. This can lead to the formation of calcium humates and fulvates, affecting the solubility and mobility of the organic matter [56].

### 3.2. Analysis of the Compressive Stress of the Composite

**Figure 2:**
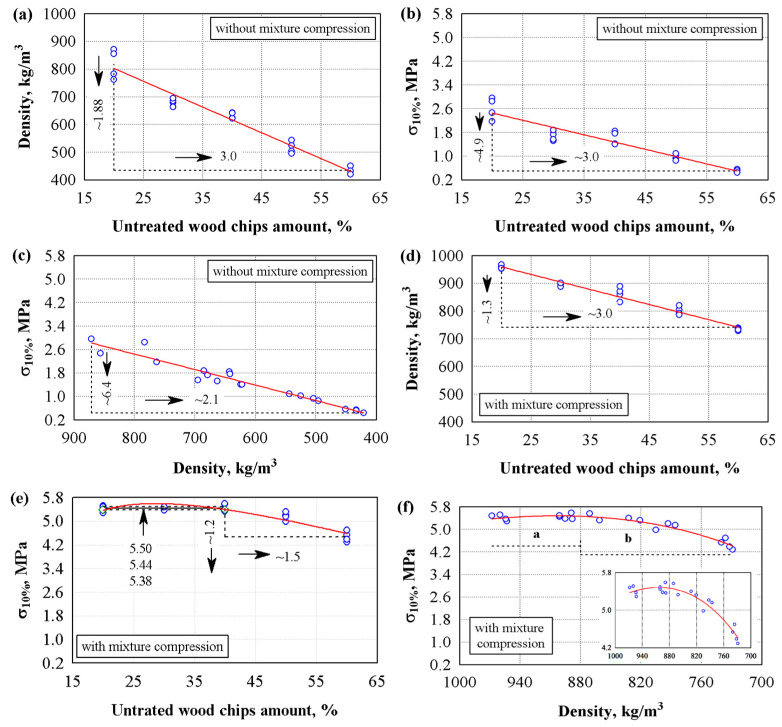
Dependence of the density and compressive stress of the composites on the amount of chemically untreated wood chips and additional coating of the mixture when the sapropel/gypsum ratio is 30/70: (**a**–**c**) specimens without mixture compression; (**d**–**f**) specimens with mixture compression; ○—experimental data; ─── average line.

It was determined how the amount of wood chips and the mixture’s additional compression affected the hardened composite’s density and compressive stress (Figure 2). The analysis showed that as the amount of wood chips increases from 20 to 60%, the density and compressive stress of the specimens decrease. That is, when the amount of wood chips increases by three times, we see that the properties of the specimens decrease linearly: density decreases up to ~1.88 times (Figure 2a), the compressive stress up to 4.9 times (Figure 2b), and when the density of the material decreases from 871 to 421 kg/m^3^ (2.1 times), compressive stress decreased by a factor of 6.4 (Figure 2c). In summary, the density of the composite decreases by an average of 100 kg/m^3^ when an additional 10% of wood chips is added, but the compressive strength decreases by about 0.5 MPa.

Researchers have determined that fillers decrease the compressive stress of composites [41,42]. A sharp decrease in strength occurs with a small amount of filler, and with a further increase in filler amount, the decrease in strength is significantly less. In our work, when we used 20% of the filler, compressive stress decreased almost four times, and when the filler was increased by 10%, the strength decreased by approximately 5%.

Figure 2d–f show the results of the test specimens formed by compressing the mixture and using chemically untreated chips to prepare the mixture. The analysis of the results shows that a 3 times increase in the amount of wood chips results in a ~1.3 times decrease in the composite density (see Figure 2d). Meanwhile, with an increase in the amount of wood chips from 20 to 40%, the average compressive stress values do not differ (see Figure 2e). The analysis of variance showed that as the content of wood chips increased from 20 to 40%, the statistic of the F-criterion was 0.38 and *p* = 0.70, indicating a statistically insignificant difference in the test results. Consequently, the coefficient of determination R^2^ = 0.078 and the corrected coefficient of determination R^2^ = 0.13 were obtained. The analysis showed that by introducing wood chips in 20 to 40% of the gypsum content, the density of the samples decreased from 970 to 900 kg/m^3^, while the compressive strength remained unchanged. This means that we can use a larger amount of wood chips without reducing the compressive strength of the composite. A further increase in wood chips from 40 to 60% (1.5 times) reduced the compressive stress by ~1.2 times.

Meanwhile, the dependence of compressive stress on density (Figure 2f) showed two zones of compressive stress. As the density decreases from ~968 to ~880 kg/m^3^, the compressive stress practically does not change. Meanwhile, as the density decreased from ~880 to ~729 kg/m^3^, the compressive stress decreased by 1.24 times. It can be assumed that the compression of the mixture leads to an increase in the compressive stress values when comparing specimens of the uncompressed and compressed mixtures. On the test path of ~880 kg/m^3^, the difference in compressive stress reaches ~1.9 times. Table 7 presents the regression equations for the resulting dependencies; see Equations (4), (7), (8), (9) and (10).

**Figure 3:**
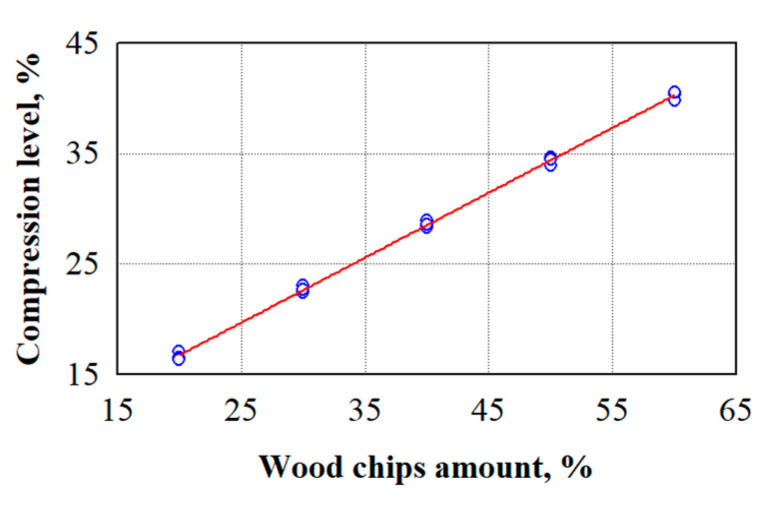
Compression level for different amounts of wood chips; ○—experimental data; ─── average line.

To reduce the effect of wood waste on strength indicators, we used two additional technological processes: the initial compression of the mixture and the chemical treatment of wood chips. Compacting the mixture is difficult because too much compaction releases water from the mixture and washes out the binding material. For this purpose, we determined the compression level for each amount of wood chips (see Figure 3). The compression level is reached when water escapes from the specimen.

The analysis of the experimental studies on the evaluation of the compression level showed a statistically significant difference in the results obtained; the statistic of the F-criterion was 8625 and *p* = 0. The dependence of the compression level on the amount of wood chips can be described by the following regression expression, Equation (1):

(1) $$C_{l}=5.0+0.5883\cdot w_{a}$$

$C_{l}—compression\;level$.

Other authors who studied the strength indicators of the gypsum composites did not compress the mixture [39,41,42,43,44]. This may be due to the rapid hardening of the gypsum and the release of water from the mixture or a small number of fillers when the compression is insignificant. Sapropel allows for controlling the setting time of the gypsum, which allows for applying compression to the composite mixture when a large amount of filler is used.

**Figure 4:**
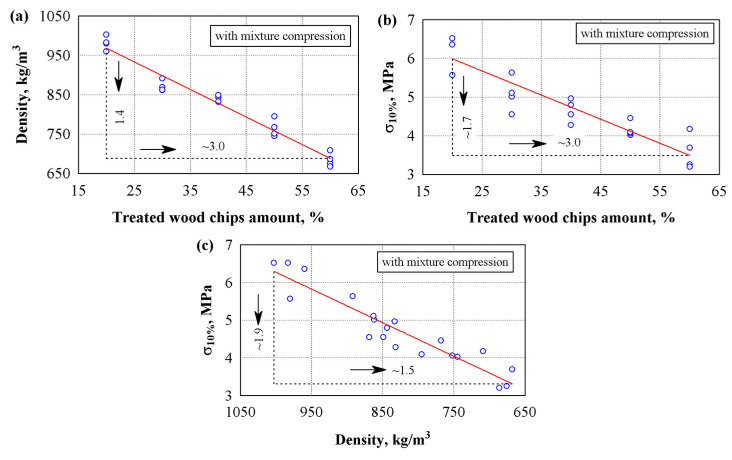
Dependencies of density and compressive stress of specimens prepared from compressed mixture and chemically treated wood chips with a sapropel/gypsum ratio of 30/70: (**a**)—density on wood chips amount; (**b**)—compressive stress on wood chips amount; (**c**)—compressive stress on density; ○—experimental data; ─── average line.

Chemically treated wood chips with sodium carbonate lost up to 30% of their mass due to the washing out of fine and soluble particles. Loss of mass from wood chips led to changes in the density and strength indicators of the composite. Figure 4 shows the dependence of the physical properties of specimens made from compressed mixture and chemically treated chips. Table 7 presents the regression equations for the resulting dependencies; see Equations (4),(7) and (8). The analysis of the experimental results shows that when the amount of wood chips increases by 3 times, the properties of the specimens decrease linearly: the density of the specimens decreases by 1.4 times (Figure 4a) and the compressive stress by 1.7 times (Figure 4b). The dependence of compressive stress and density showed that when the density decreased from ~1003 to 668 kg/m^3^, the compressive stress decreased by ~1.9 times (Figure 4c).

Comparing the compression values shown in Figure 2e and Figure 4b, when 60% of wood chips are used, it is shown that the compressive stress of the specimens with treated chips has decreased. The chemical treatment likely weakened the wood particle strength but improved the bond with the binder. When comparing the compressive strength of a composite made from 20–30% washed or unwashed wood chips, the values are either not different or slightly different. Using 40%, the strength decreases by 13%, and using 60%, this decreases by 23%. In the works of other authors, wood treatment was carried out with water of different temperatures and Na(OH) solution [33,44]. The authors found that additional thermal and chemical treatment improves the strength properties of the composite.

**Figure 5:**
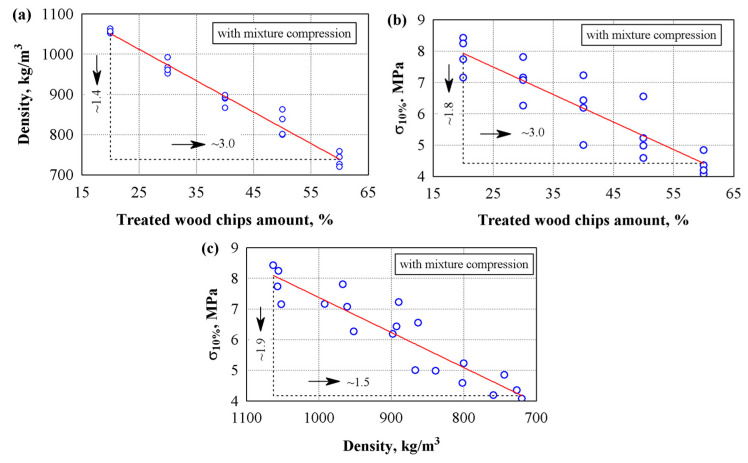
Dependencies of density and compressive stress of specimens prepared from compressed mixture and chemically treated wood chips when gypsum and lime are used only: (**a**)—density on wood chips amount; (**b**)—compressive stress on wood chips amount; (**c**)—compressive stress on density; ○—experimental data; ─── average line.

Figure 5a–c show the dependence of the physical properties of specimens prepared from a compressed mixture with chemically treated wood chips when only gypsum and lime are used. Table 7 presents the regression equations for the resulting dependencies; see Equations (4), (7) and (8). The analysis of the experimental results showed that when the amount of wood chips increases by 3 times from 20 to 60%, the properties of the specimens decrease linearly: the density of the specimens decreases by 1.4 times, and the compressive stress decreases by 1.8 times. The dependence of compressive stress and density showed that when the density decreased from ~1063 to 720 kg/m^3^, the compressive stress decreased by ~1.9 times.

Comparing the results between the binding materials used for composites with chemically treated wood chips, sapropel/gypsum/lime, and gypsum/lime, we see that the density of the composite increases from 7 to 10%, and the compressive strength increases from 20 to 24% when the binding material of the composite is only gypsum and lime.

### 3.3. Analysis of the Thermal Conductivity

**Figure 6:**
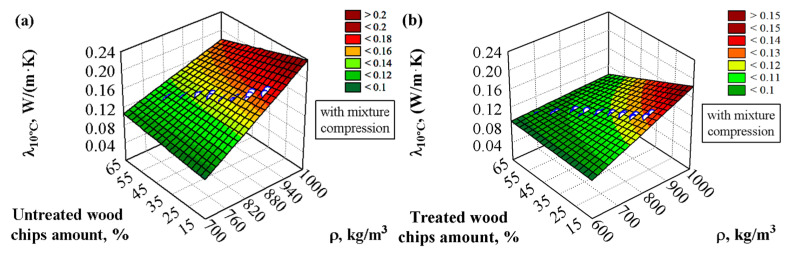
Dependence of the thermal conductivity on the density of the specimens and the amount of wood chips when the binding material consists of gypsum, sapropel, and lime, and the filler is wood chips: (**a**) chemically untreated, (**b**) chemically treated; ○–experimental data.

**Figure 7:**
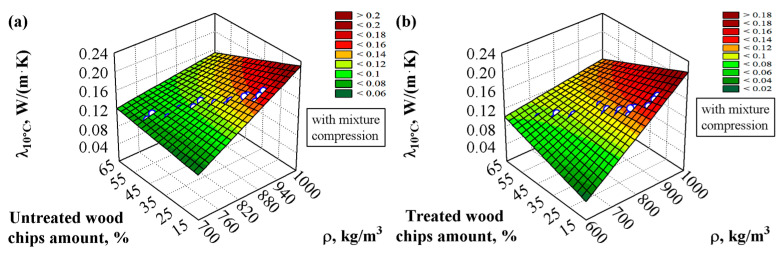
Dependence of the thermal conductivity on the density of the specimens and the amount of wood chips when the binding material consists of gypsum and lime and the filler is wood chips: (**a**) chemically untreated, (**b**) chemically treated; ○–experimental data.

The compressive strength deteriorates when the sapropel is used, but the thermal insulation properties improve. Multi-criteria regression analysis was applied to process the experimental measurement results to estimate the thermal conductivity of the composite material more accurately. After a mathematical–statistical analysis was performed, it was possible to calculate the values of the thermal conductivity based on the obtained results of the density and amount of wood chips. The graphical expression of the results of the experimental thermal conductivity values is shown in Figure 6a,b and Figure 7a,b, and the statistical processing results are shown in Table 8, Equation (11).

The analysis showed that the processing of the wood chips greatly influences the thermal conductivity values. It can be seen that using chemically treated wood chips results in lower thermal conductivity values than chemically untreated chips. Also, the amount of wood chips in the material reduces the thermal conductivity. The higher the amount of chips, the lower the thermal conductivity value. In addition, the thermal conductivity is lower in all cases when the sapropel is used (Figure 6) compared to the composites when the sapropel is not used. Compared to composites prepared from untreated chips and an uncompressed mixture, the thermal conductivity decreased by about 25%. A lower coefficient of thermal conductivity allows for the use of a smaller amount of material to achieve the required thermal resistance. The most important property of such a composite is thermal conductivity, which can be used as a thermal insulation or a thermal insulation structural material.

Other scientists have also examined the influence of fillers on thermal conductivity [39,57]. Ejaz et al. [57] found that using 10% filler gives a thermal conductivity of 0.127 W/(m·K). This result resembles our work when the binding material is only gypsum and lime. Then, Vavrinova et al. [39] found that 5% straw filler had almost no effect on thermal conductivity reductions.

### 3.4. Analysis of the Microstructure

When the properties of composite material are studied, the structure’s peculiarities determine the material’s physical properties and the dispersion of the experimental values obtained. A binder with a different composition has a different structure and formation when hardened, which also changes the properties of the composite.

**Figure 8:**
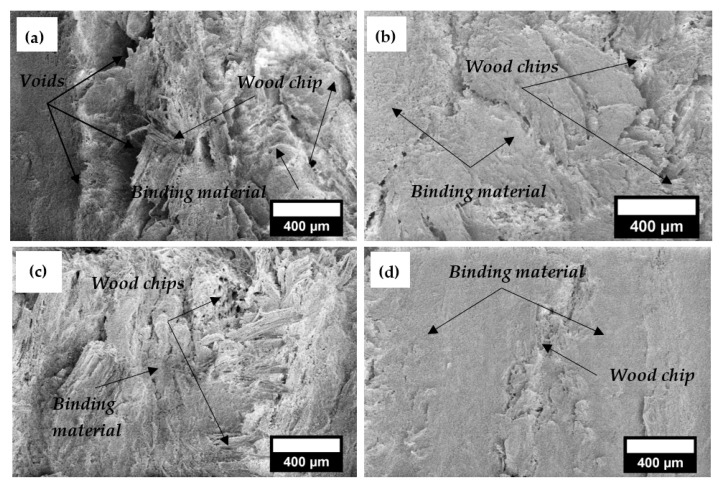
SEM images of composites (magnification ×100): (**a**) composite mixture of gypsum (70%), sapropel (30%), lime (10%), and wood chips (untreated) via a manual compaction of mixture; (**b**) binding material the same as in (**a**) via a mixture compacted by compression; (**c**) binding material the same as in (**a**) but with treated wood chips via a mixture compacted by compression; (**d**) composite mixture of gypsum (70%), lime (10%), and wood chips (treated) via a mixture compacted by compression.

Figure 8 shows a general view of the composites with 50% of wood chips. Structural analysis shows that a specific structure is formed in differently prepared composites. When the compression of the mixture is not used (Figure 8a), voids are observed between the wood chips and the binder. Figure 8b,c, and d show that when mixtures of composites are compressed, large voids do not remain in the structure of the composites. Due to the heterogeneous structure of the composite, which contains voids and poor adhesion between the binding material and wood chips, the compressive strength of the composite is reduced, and the thermal conductivity deteriorates.

**Figure 9:**
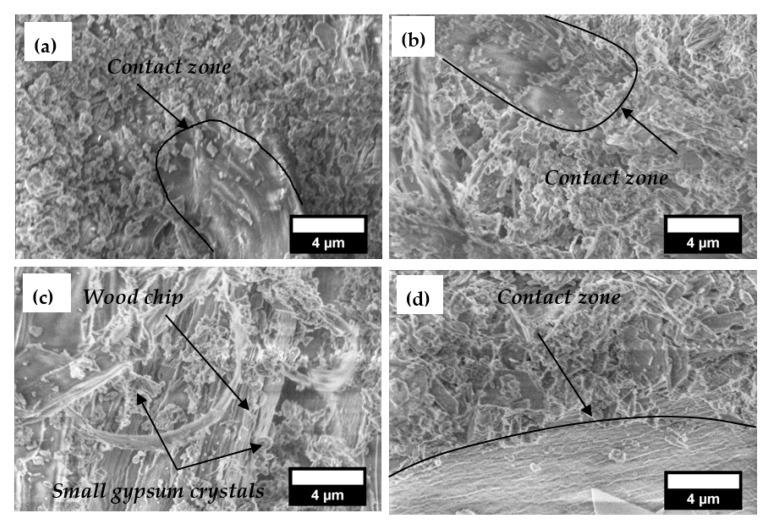
Contact zone analysis between the binding material and the filler, ×10,000: (**a**) composite mixture of gypsum (70%), sapropel (30%), lime (10%), and wood chips (untreated) via a manual compaction of mixture; (**b**) binding material the same as in (**a**) via a mixture compacted by compression; (**c**) binding material the same as in (**a**) but with treated wood chips via a mixture compacted by compression; (**d**) composite mixture of gypsum (70%), lime (10%), and wood chips (treated) via a mixture compacted by compression.

Figure 9 analyses the contact zones between the binder and the wood chips. When the composite mixture is not compressed and untreated wood chips are used, individual binder particles or short crystals come into contact with the filler (Figure 9a). When the composite mixture is compressed and untreated wood chips are used, there are almost no individual binder particles in contact with the filler, and smaller and larger crystals are formed (Figure 9b). When the composite is compressed and treated wood chips are used, crystals are formed that are mostly interconnected of various sizes (Figure 9c). When the composite mixture is compressed, treated wood chips are used, and the binder is only gypsum and lime. Plates of various sizes are formed in the filler, overlapping in the contact zones (Figure 9d).

In the work of Kairytė et al. [58], it was found that untreated wood chips do not provide good contact areas between wood particles and the binding material. Poor compatibility, deficient bonding between wood chips and binding material, and voids were observed between the wood chips and the binding material matrix.

Tuğluc et al. [59] have found that chemically treated wood particle fibres become coarser, provide better adhesion to the binder, and do not form gaps between the binder matrix and the filler. In our work, the chemical treatment of wood chips and additional compression allowed for the creation of reliable contact zones between wood chips and binding material.

## 4. Conclusions

Using sapropel extends the start of the mixture’s hardening and improves the composite’s properties, reducing its density by 10% and thermal conductivity by 25%.

The use of organic filler significantly reduces the density and thermal conductivity of the composite but worsens its strength characteristics. The composite density decreases with untreated wood chips with no compression from 800 to 430 kg/m^3^, i.e., 47%. With untreated wood chips and compression, it decreases from 960 to 740 kg/m^3^, i.e., 33%, and with treated wood chips and compression, it decreases from 970 to 670 kg/m^3^, i.e., 31%. The thermal conductivity of the composite decreases with untreated wood chips and with compression, from 0.193 to 0.114 (W/m·K), i.e., 41%, and with treated wood chips and with compression, from 0.146 to 0.0912 (W/m·K), i.e., 38%.

Compressing the mixture, chemically treating wood chips, and using 10% hydrated lime can significantly increase the compressive stress. These factors increase the composite’s compressive strength from 2.9 to 6.0 MPa by volume, or 90%.

When using more than 40% wood chips, their chemical treatment weakens the strength of the wood particles, which also slightly weakens the strength of the composite. However, chemical treatment improves adhesion to the binder, which allows for an improvement of up to 25% in the thermal properties of the composite.

Using sapropel and lime to modify gypsum created different bonds, while further compressing the mixture allowed for closer contact zones between the wood chips and the binding material.

The research results show that the maximum amount of filler, which allows the obtaining of a composite with low density and thermal conductivity and does not deteriorate the strength characteristics, is 40%.

Such material can be used to insulate building partitions, construct frames, and produce blocks and panels.

## Figures and Tables

**Table 1:** Main characteristics of the sapropel.

Sampling Place of Sapropel	Water Content, Mass %	Acidity (pH)	Content of Organic Matter, Mass %
Lake Gervinis, the District of Alytus, Lithuania	93.0	5.7–7.4	92.0

**Table 2:** Characteristics of gypsum.

Content of Water, %	Consistency, mm	Initial Setting Time, min	Final Setting Time, min	Density of Dry Hardened Gypsum, kg/m^3^	Compressive Strength of Dry Hardened Gypsum, MPa
60	160	10	15	1350	9.5

**Table 3:** Granulometric composition of wood chips.

The Size of the Sieve Mesh, mm						
10	5	2.5	1.25	0.63	0.315	0
**Residues of Wood Chips on Sieves, wt.%**						
1.5	38.8	50.8	3.0	4.3	1.3	0.3

**Table 4:** Compositions of specimens for compressive strength determination of binding material (see Figure 1).

Sapropel, %	Gypsum, %	Sapropel/ Gypsum	Lime, % *	Lime, r. u. **	Water, % ***	
					Water from Sapropel	Total Water Content
10 20 30 40 50 60 70 80	90 80 70 60 50 40 30 20	0.11 0.25 0.43 0.67 1.00 1.50 2.33 4.00	–	–	30	60
30	70	0.43	5; 10; 20	0.05; 0.10; 0.20		
35	65	0.54	5; 10; 20	0.05; 0.10; 0.20		
40	60	0.67	5; 10; 20	0.05; 0.10; 0.20		
45	55	0.82	5; 10; 20	0.05; 0.10; 0.20		
50	50	1	5; 10; 20	0.05; 0.10; 0.20		

* The amount of lime was calculated as a percentage of gypsum. ** r. u.-relative units. *** The structure of sapropel contains 93% water, part of which is easily released during the reaction with gypsumt. When calculating the total amount of water required for the hardening of gypsum, 30% of the water is calculated as coming from the sapropel structure.

**Table 5:** Compositions of specimens for compressive strength determination of composite material (see Figure 2, Figure 3, Figure 4 and Figure 5).

Sapropel, %	Gypsum, %	Lime, % *	Water, %		Wood chips, %	
			Water from Sapropel	Total Water Content	Chemically Untreated	Chemically Treated
30	70	10	30	80	20; 25; 30; 35; 40; 45; 50; 55; 60	–
30	70	10	30	60	20; 25; 30; 35; 40; 45; 50; 55; 60	–
30	70	10	30	60	–	20; 25; 30; 35; 40; 45; 50; 55; 60

* The amount of lime was calculated as a percentage of gypsum.

**Table 6:** Compositions of specimens for the measurement of thermal conductivity of composite material (see Figure 6 and Figure 7).

Sapropel, %	Gypsum, %	Lime, % *	Water, %		Wood chips, %	
			Water from Sapropel	Total Water Content	Chemically Untreated	Chemically Treated
30	70	10	30	60	20; 25; 30; 35; 40; 45; 50; 55; 60	–
30	70	10	30	60	–	20; 25; 30; 35; 40; 45; 50; 55; 60
–	100	10	–	60	20; 25; 30; 35; 40; 45; 50; 55; 60	–
–	100	10	–	60	–	20; 25; 30; 35; 40; 45; 50; 55; 60

* The amount of lime was calculated as a percentage of gypsum.

**Table 7:** Regression equations for composite physical values.

No.	Equations of Composite Characteristics	Units
(2)	$\rho =b_{0}+exp(b_{1}+b_{2}\cdot s/g$)	kg/m^3^
(3)	$\sigma_{10\%}=b_{0}+exp(b_{1}+b_{2}\cdot$*s*/*g*)	MPa
(4)	$\sigma_{10\%}=b_{0}+b_{1}\cdot \rho$	MPa
(5)	$\rho =b_{0}+b_{1}\cdot s/g+b_{2}\cdot l+b_{3}\cdot {\left(s/g\right)}^{2}$	kg/m^3^
(6)	$\sigma_{10\%}=b_{0}+b_{1}\cdot s/g+b_{2}\cdot l+b_{3}\cdot {\left(s/g\right)}^{2}+b_{4}\cdot l^{2}$	MPa
(7)	$\rho =b_{0}+b_{1}\cdot w_{a}$	kg/m^3^
(8)	$\sigma_{10\%}=b_{0}+b_{1}\cdot w_{a}$	MPa
(9)	$\sigma_{10\%}=b_{0}+b_{1}\cdot w_{a}+b_{2}\cdot w_{a}$	MPa
(10)	$\sigma_{10\%}=b_{0}+b_{1}\cdot \rho +b_{2}/\rho$	MPa
(11)	$\lambda_{10{}^{\circ}C}=b_{0}+b_{1}\cdot \rho +b_{2}\cdot w_{a}\cdot b_{3}\cdot \rho \cdot w_{a}$	W/(m·K)

NOTE: $\rho —{materials}^{’}density,\;{kg/m}^{3};$ $b_{0},\;b_{1},\;b_{2},\;b_{3,\;}b_{4}—the\;values\;of\;coefficients\;of\;regression\;analysis;$ $\sigma_{10\%}—$compressive stress at 10% deformation, MPa; $w_{a}—wood\;chips\;amount,\%;\;l—lime,\%;\;sapropel,\%;\;g—gypsum,\%$.

**Table 8:** Statistical results of physical properties of composites.

Equation No./Figure No.	No. of Specimens		Statistical Characteristics									
		$b_{0}$	$b_{1}$	$b_{2}$	$b_{3}$	$b_{4}$	R	R^2^	*R^2^	$S_{r}$	F	***p***
2/1a	37	−0.1	6.531	−0.912	–	–	0.995	0.989	0.988	21.5	15,033	0
3/1b	37	0.01373	−0.1023	−0.8696	–	–	0.968	0.937	0.933	0.071	731	0
4/1c	37	−0.4631	0.00130	–	–	–	0.971	0.942	0.940	0.067	568	0
5/1d	60	1006.755	−468.139	434.79	81.07	–	0.980	0.961	0.958	15.8	388.8	0
6/1e	60	1.23829	−1.10564	2.44309	0.30147	−6.66552	0.888	0.789	0.773	0.0782	51.3	0
4/1f	60	−0.6597	0.00188	–	–	–	0.896	0.803	0.799	0.074	236	0
7/2a	20	988.950	−9.3075	–	–	–	0.976	0.953	0.951	30.7	369	0
8/2b	20	3.4347	−0.0489	–	–	–	0.943	0.890	0.884	0.26	146	0
4/2c	20	−1.7808	0.00528	–	–	–	0.971	0.944	0.941	0.18	302	0
7/2d	20	1068.35	−5.440	–	–	–	0.983	0.967	0.965	14.9	531	0
9/2e	20	9.09291	−0.06130	−50.0117	–	–	0.911	0.830	0.798	0.17	45.6	0
10/2f	20	51.6786	−0.02557	−20864	–	–	0.960	0.922	0.907	0.12	105	0
7/4a	20	1108.45	−7.000	–	–	–	0.979	0.959	0.956	21.7	417	0
8/4b	20	7.23665	−0.06235	–	–	–	0.919	0.844	0.835	0.40	97.4	0
4/4c	20	−2.6382	0.00891	–	–	–	0.939	0.881	0.874	0.35	133	0
7/5a	20	1207.50	−7.8100	–	–	–	0.988	0.976	0.974	18.3	726	0
8/5b	20	9.6895	−0.08781	–	–	–	0.900	0.810	0.799	0.64	76.5	0
4/5c	20	−4.0518	0.01143	–	–	–	0.926	0.858	0.849	0.55	108	0
11/6a	27	−0.3333	0.591·10^−3^	0.004102	−0.53·10 ^−5^	–	0.988	0.976	0.971	0.0035	310	0
11/6b	27	−0.1059	0.000315	0.002726	−0.44·10 ^−5^	–	0.988	0.977	0.973	0.0027	328	0
11/7a	27	−0.2827	0.536·10^−3^	0.00505	−0.66·10 ^−5^	–	0.981	0.963	0.956	0.053	201	0
11/7b	27	−0.3048	0.546·10^−3^	0.005416	−0.70·10 ^−5^	–	0.979	0.959	0.951	0.0048	182	0

NOTE: The F criterion is a value used in statistics to test hypotheses; the *p*-value is the probability of observing test results; *Sr* is the standard deviation; R is the correlation coefficient; R^2^ is the coefficient of determination; *R^2^ is the corrected coefficient of determination.

## Data Availability

The original contributions presented in this study are included in the article. Further inquiries can be directed to the corresponding author.
